# ELDER VOICES NETWORK: PARTNERING WITH OLDER PEOPLE AND THEIR CAREGIVERS TO AVOID MEDICAL HARM

**DOI:** 10.1093/geroni/igz038.2544

**Published:** 2019-11-08

**Authors:** Molly Ranahan, Mary Brennan-Taylor, Michael Richbart, Collin Clark, Ryan Gadzo, Ranjit Singh, Robert Wahler

**Affiliations:** 1 University at Buffalo, Buffalo, New York, United States; 2 Erie County Department of Senior Services, Buffalo, New York, United States

## Abstract

Team Alice, named after an older adult in our community who died as a result of medication harm, is an interdisciplinary team of prescribers, pharmacists, educators, advocates, and researchers with a mission to protect older people from medication-related harm across the continuum of care. In 2019, Team Alice partnered with the Erie County Department of Senior Services, older people, and caregivers to form the Elder Voices Network (EVN) as a vehicle for patient-driven deprescribing in the Western New York region. The objective of this presentation is to detail the planning and implementation of critical components of EVN’s formation, including outreach and engagement, funding development, community partnerships, roles and communication, and decision-making. Case study results demonstrate the capacity of community-based participatory research (CBPR) to empower older people and caregivers with knowledge, skills, and tools to promote self-advocacy across the system. Presenters will also discuss recommendations useful for future patient engagement initiatives.

